# Characteristic MRI pattern in *LMNB1*-related autosomal dominant leukodystrophy: a case report

**DOI:** 10.3389/fnins.2026.1821432

**Published:** 2026-06-03

**Authors:** Yu-Xin Wang, Tong Du, Chun-Lin Yang, Min Zhang, Rui-Sheng Duan

**Affiliations:** 1 Department of Neurology, The First Affiliated Hospital of Shandong First Medical University and Shandong Provincial Qianfoshan Hospital, Jinan, China; 2 Shandong Institute of Neuroimmunology, Jinan, China; 3 Shandong Provincial Medicine and Health Key Laboratory of Neuroimmunology, Jinan, China

**Keywords:** adult-onset autosomal dominant leukodystrophy, Beagle sign, case report, *LMNB1* gene, MRI

## Abstract

**Introduction:**

Adult-onset autosomal dominant leukodystrophy (ADLD) is an ultra-rare inherited white matter disorder caused by variants in the *LMNB1* gene. Here, we report a case of ADLD and characterize its typical magnetic resonance imaging (MRI) features, with the aim of facilitating its clinical recognition and differential diagnosis.

**Case description:**

The patient was a 55-year-old male who had experienced incomplete voiding, dysuria, and constipation for 10 years. One year prior to presentation, he developed lower limb weakness and unsteady gait, which progressively worsened over time. Brain MRI revealed extensive white matter abnormalities, including a symmetric hyperintensity pattern in the brainstem corticospinal tract and bilateral middle cerebellar peduncles on T2-weighted/FLAIR images, which resembled the facial profile of a Beagle dog. Subsequent genetic testing identified a pathogenic duplication of the *LMNB1* gene, a typical variant associated with ADLD.

**Conclusion:**

We report a case of ADLD caused by *LMNB1* duplication with a typical clinical course and characteristic MRI features. Its characteristic MRI features, including the “Beagle sign” as an illustrative imaging analogy in the brainstem, may facilitate the clinical recognition and differential diagnosis of this disorder.

## 1 Introduction

Adult-onset autosomal dominant leukodystrophy (ADLD) is an ultra-rare inherited white matter disorder caused by variants in the *LMNB1* gene (Raininko et al., 1993). The disease is characterized by autonomic dysfunction, motor impairment, and cerebellar ataxia, which result from white matter dystrophy in the brain and spinal cord, including the corticospinal tracts and middle cerebellar peduncles (Guaraldi et al., 2011; Finnsson et al., 2015; Terlizzi et al., 2016). The clinical manifestations of ADLD are diverse, with symptoms gradually worsening over time. With advances in genetic testing, more ADLD patients are being diagnosed, but imaging features remain a crucial basis for diagnosis. Traditional imaging findings include symmetrical white matter lesions, particularly in the subcortical white matter, brainstem, and middle cerebellar peduncles (Finnsson et al., 2015; Sundblom et al., 2009; Melberg et al., 2006). On T2-weighted or FLAIR sequences, the symmetrical hyperintensities in the brainstem corticospinal tract and bilateral middle cerebellar peduncles resemble the profile of a Beagle dog. So, to assist in the visual identification of these established patterns, we utilize a Beagle sign analogy to characterize the symmetric hyperintensities in the brainstem and middle cerebellar peduncles, thereby facilitating the recognition of such features in clinical practice.

## 2 Case report

### 2.1 Patient history

The patient was a 55-year-old male who had experienced incomplete voiding, dysuria, and constipation for 10 years. He was diagnosed with benign prostatic hyperplasia and underwent surgical treatment 9 years ago, but with unsatisfactory outcome. One year prior to presentation, he developed lower limb weakness, unsteady gait, numbness in the soles of both feet, a cotton-like sensation when walking, and coldness in both lower limbs, which progressively worsened over time. He had a 30-year history of alcohol consumption, averaging half a pound of baijiu (Chinese liquor) per day, and had abstained from alcohol for 1 year. He also had a 30-year smoking history, with 20 cigarettes per day, and denied any history of sexually transmitted diseases or drug use.

### 2.2 Neurological examination

Neurological examination on presentation revealed that the patient’s speech was somewhat unclear, with an abnormal gait characterized by a wide-based stance and impaired stride length bilaterally. The Romberg test was negative, but the sharpened Romberg test was positive. The tandem walking test showed unsteadiness, and there was impairment in recent memory. Bilateral patellar tendon reflexes were symmetrically hyperactive (3+), accompanied by positive bilateral Hoffmann signs. Muscle strength in all four limbs was normal. Sensory examination revealed normal light touch and pinprick sensations in the limbs and face. Vibration sense was normal in both upper limbs but reduced in both lower limbs. The finger-to-nose test showed mild impairment bilaterally, and the heel-to-shin test was unsteady and imprecise bilaterally. Routine laboratory tests, including vitamin and homocysteine levels, were unremarkable. Ultrasound examination of both lower limbs revealed left common femoral vein valve insufficiency, with no other significant abnormalities detected, which could not explain the symptoms of coldness and weakness in both lower limbs. Motor and sensory nerve conduction studies showed no significant abnormalities in the lower limbs. Somatosensory evoked potentials of the lower limbs exhibited well-differentiated waveforms, with prolonged P38 latency. Bilateral anal sphincter muscles showed no spontaneous electrical activity at rest.

### 2.3 Imaging findings

**Figure 1:**
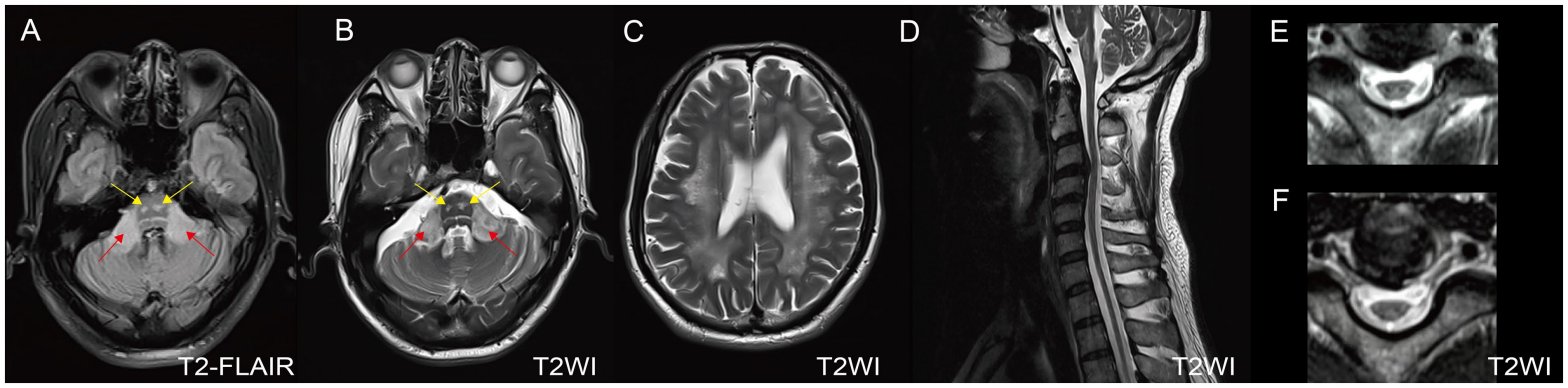
MRI images demonstrating characteristic findings in ADLD. The corticospinal tract in the brainstem (yellow arrow) and middle cerebellar peduncles (red arrow) show bilateral symmetric hyperintensity in T2 FLAIR **(A)** and T2-weighted **(B)**, resulting in the “Beagle sign” as an illustrative imaging analogy. Symmetric diffuse hyperintensity in the centrum semiovale and periventricular white matter of the frontal and parietal lobes on T2-weighted **(C)**. Sagittal **(D)** and representative axial **(E,F)** T2-weighted cervical spinal MRI reveal diffuse spinal cord atrophy.

The patient’s brain MRI revealed symmetrical and confluent cerebral white matter hyperintensities on T2-weighted images. T2 hyperintensities affected the frontal, parietal and occipital lobes, and signal intensity of periventricular white matter was lower than that of more peripheral white matter. The pyramidal tracts showed diffuse T2 hyperintensity along their entire course, including the cerebral peduncles, ventral pons, and pyramids of the medulla oblongata. The white matter hyperintensities in the brainstem and bilateral middle cerebellar peduncles form a characteristic pattern that can be referred to as the “Beagle sign” as an illustrative imaging analogy: the abnormal signal in the brainstem corticospinal tract resembles the eyes of a beagle dog, while the hyperintensities in the bilateral peduncles resemble its large ears (Figure 1). The same signal pattern was observed on T2-weighted FLAIR images. On DWI sequences, similar extensive hyperintensities were observed in the white matter, with a higher DWI signal in the juxtacortical areas. ADC values in the affected areas are not decreased. Cervical and spinal MRI further revealed a diffuse demyelinating process, with spinal cord atrophy. Arterial spin labeling (ASL) perfusion imaging demonstrated regions of decreased regional perfusion within the periventricular white matter and pons. This pattern likely reflects reduced metabolic activity, consistent with a chronic neurodegenerative process rather than acute inflammatory changes. Magnetic resonance spectroscopy (MRS) identified corresponding metabolic abnormalities, including an elevated lactate peak. While these imaging and metabolic alterations offer supportive clues for further characterization, their specificity and definitive diagnostic value in leukodystrophy remain to be fully established. The overall imaging findings are highly suggestive of *LMNB1*-related autosomal dominant leukodystrophy, whose typical MRI features include frontoparietal white matter lesions, brainstem and middle cerebellar peduncles hyperintensities as well as spinal cord atrophy, providing important diagnostic clues.

### 2.4 Gene detection

The patient initially underwent whole-exome sequencing (WES), which suggested a suspected 141.09 kb duplication at 5q23.2 (chr5:126112828–126253919, hg19). To confirm this finding and evaluate genetic segregation, both the patient and his son (who was asymptomatic at the time) underwent high-throughput whole-genome sequencing (NGS-CNV-seq). This orthogonal verification precisely identified an identical 280 kb duplication at the same 5q23.2 locus (chr5:126040001–126320000, hg19) in both individuals. The duplicated region encompasses the triplosensitive gene *LMNB1*, and the variant was classified as pathogenic according to the ACMG-CNV guidelines. This genetic evidence confirms the inheritance of the pathogenic duplication within the family, providing a definitive molecular diagnosis for ADLD.

### 2.5 Follow-up

**Figure 2:**
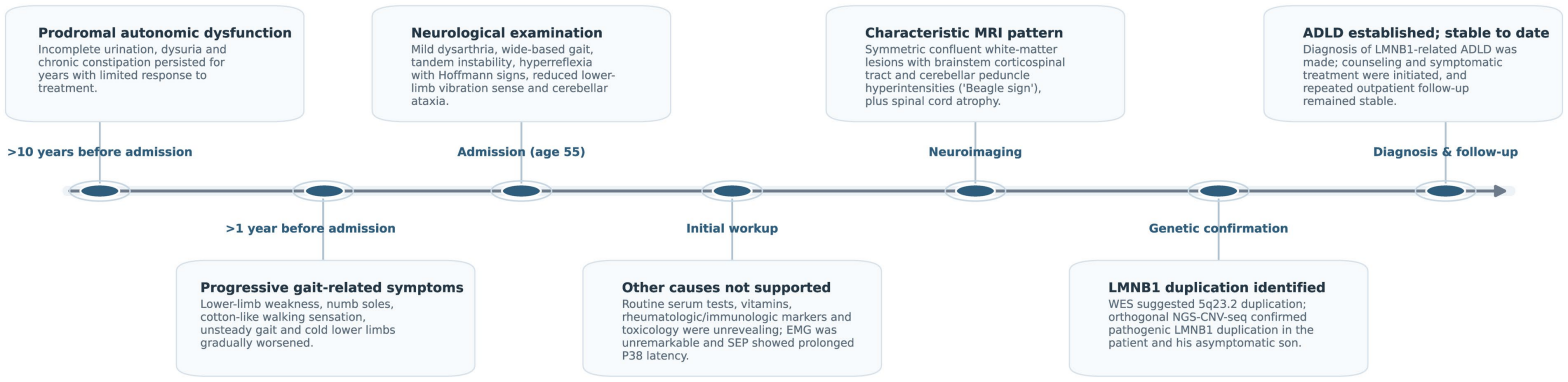
Horizontal timeline summarizing symptom evolution, diagnostic workup, neuroimaging findings, and follow-up.

**Figure 3:**
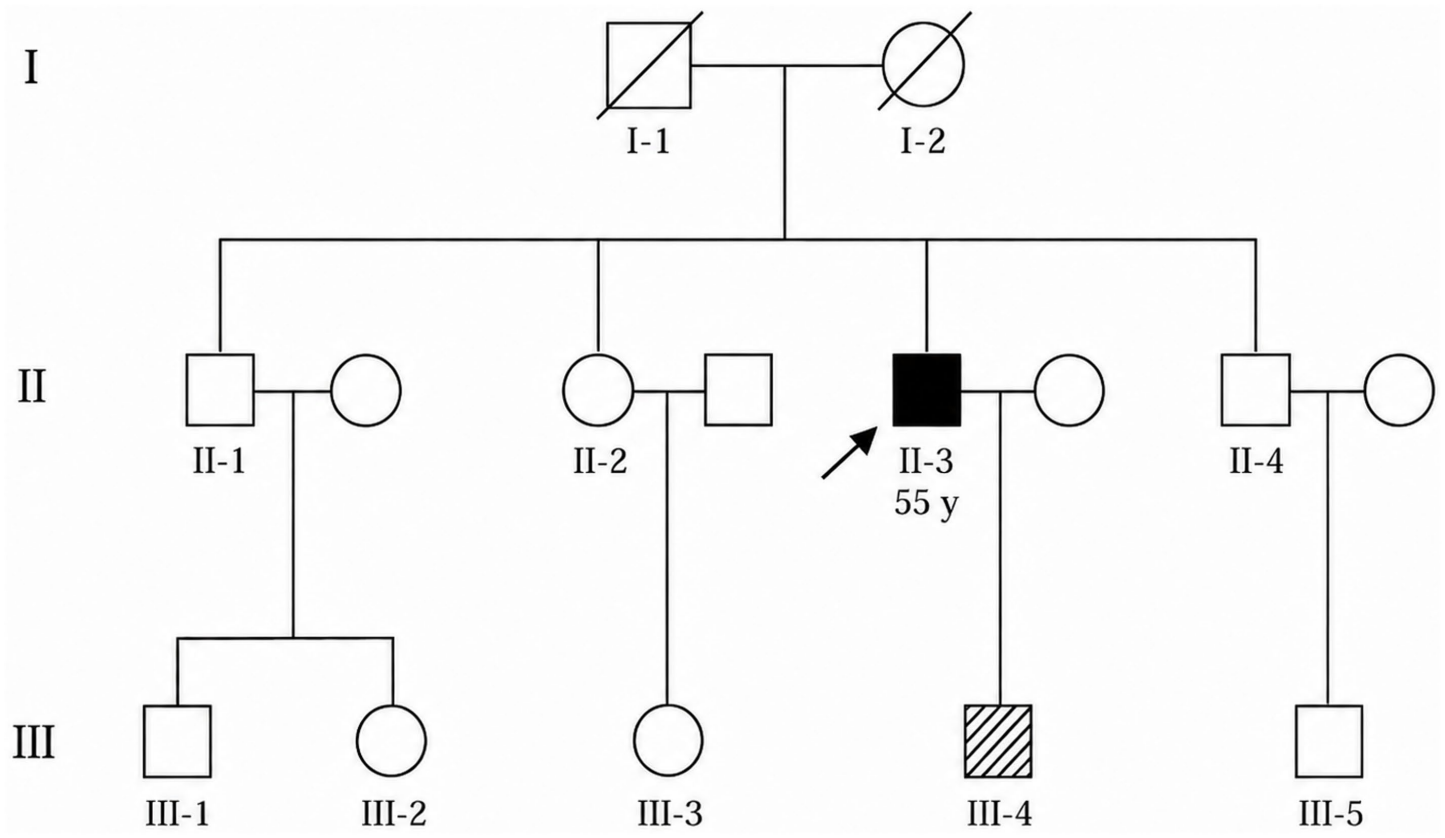
Family pedigree of adult leukodystrophy patients with autosomal dominant inheritance. Squares indicate males, and circles indicate females. Open symbols indicate unaffected individuals without the ADLD mutation. Filled black symbols indicate affected individuals carrying the ADLD mutation. The hatched square indicates an asymptomatic male who is positive for the ADLD mutation. A diagonal slash indicates a deceased individual. The arrow indicates the proband. In this family, only the proband, II-3, and his son, III-4, carry the ADLD mutation; all other family members, including the proband’s spouse and the offspring of his siblings, are unaffected and mutation-negative.

Based on the clinical presentation and diagnostic findings, a definitive diagnosis of ADLD was established. The patient and his family were counseled regarding the nature of the disease and its prognosis. Symptomatic management was initiated, including multivitamin supplementation, neurotrophic agents, cognitive enhancers, and stool softeners, alongside dietary guidance. Since discharge, the patient has attended multiple follow-up visits at our outpatient clinic, and his condition has remained stable to date. Clinical milestones and progression details are outlined in the comprehensive timeline (Figure 2). A three-generation family pedigree was further reviewed. No known individuals in the previous generation were reported to have similar neurological or autonomic symptoms. The proband’s son was clinically asymptomatic at the time of evaluation but was found to carry the same LMNB1 duplication. Other available family members had no known similar manifestations. A three-generation pedigree is provided in Figure 3.

## 3 Discussion

Leukodystrophy refers to a group of rare, inherited, progressive disorders caused by genetic factors that impair myelination and disrupt normal development (Van Der Knaap et al., 2019). Most leukodystrophies have an early onset during infancy or childhood and are typically inherited in an autosomal recessive or X-linked recessive manner (Ashrafi et al., 2020; Ceravolo et al., 2024). In contrast, *LMNB1*-related autosomal dominant leukodystrophy is an exceptionally rare and infrequently reported adult-onset leukodystrophy, falling within the category of dominant inherited disorders (Neri et al., 2023). Its primary clinical features include autonomic dysfunction as the initial symptom, followed by pyramidal tract dysfunction, cerebellar ataxia, sensory deficits in the medial cerebellar pathways, and slowly progressive cognitive impairment. Autonomic dysfunction is widespread and commonly manifests as constipation, bladder dysfunction, orthostatic hypotension, and erectile dysfunction (Finnsson et al., 2015; Terlizzi et al., 2016). Cognitive impairment typically appears in the advanced stages of the disease (Laforce et al., 2013). Due to its slow progression and gradual onset of symptoms, early diagnosis is often overlooked in many patients.

Although ADLD is relatively rare, its clinical manifestations and imaging characteristics have gradually been recognized. Our patient demonstrates the classic MRI triad of ADLD: (1) frontoparietal white matter lesions; (2) infratentorial involvement of the brainstem corticospinal tract and the middle cerebellar peduncles; and (3) spinal cord atrophy. These findings are consistent with previously published case series (Melberg et al., 2006; Zhang et al., 2019; Mezaki et al., 2018; Dhamija et al., 2024; Jiang et al., 2025; Dai et al., 2017). To facilitate clinical recognition, we propose the “Beagle sign” as an illustrative imaging mnemonic. On T2-weighted or FLAIR images, symmetric hyperintensities in the brainstem and bilateral middle cerebellar peduncles form a configuration resembling the facial outline of a beagle (Figure 1). This sign comprises two key anatomical components: (1) symmetric hyperintensity within the brainstem corticospinal tract; and (2) bilateral, symmetric involvement of the middle cerebellar peduncles. A retrospective review of published ADLD cases suggests that the anatomical components of the “Beagle sign” have been present in previously reported patients, although these anatomical components have not been explicitly described as a unified sign. For example, the original illustrations by Melberg et al. (2006) demonstrated involvement of the brainstem and middle cerebellar peduncles, and the Mayo Clinic series (Dhamija et al., 2024) noted “T2 hyperintensity in the brainstem and middle cerebellar peduncles in all patients.”

It should be emphasized that the involvement of the bilateral middle cerebellar peduncle’s involvement is not exclusively limited to *LMNB1*-related ADLD. Other leukoencephalopathies may also present with overlapping middle cerebellar peduncle abnormalities (Muthusamy et al., 2023). Table 1 summarizes the distinct clinical and radiological features of leukoencephalopathies with middle cerebellar peduncle involvement. LBSL, caused by pathogenic variants in DARS2, may present with cerebellar ataxia, spasticity, sensory tract involvement, and characteristic MRI abnormalities involving the cerebral white matter, corticospinal tracts, brainstem pathways, cerebellar white matter, and spinal cord; however, the spinal cord lesions in LBSL typically involve the dorsal columns and lateral corticospinal tracts, and MR spectroscopy may show lactate elevation (Engelen et al., 1993). CLCN2-related leukoencephalopathy can also involve the middle cerebellar peduncles and brainstem tracts. Crucially, however, its lesions typically exhibit restricted diffusion on diffusion-weighted imaging (DWI) secondary to intramyelinic edema. Furthermore, it is often associated with optic atrophy, retinal abnormalities, hearing symptoms, or hypogonadism, and usually lacks the typical long-standing autonomic dysfunction and spinal cord atrophy observed in LMNB1-related ADLD (Depienne et al., 2013). Fragile X-associated tremor ataxia syndrome (FXTAS) is another important mimic because the classical middle cerebellar peduncle sign may be accompanied by splenial and diffuse white matter abnormalities, but the usual clinical context is late-onset intention/action tremor, cerebellar ataxia, cognitive decline, and FMR1 premutation (Hunter et al., 1993). Neuronal intranuclear inclusion disease (NIID) may also involve the middle cerebellar peduncles and diffuse white matter, but the presence of curvilinear diffusion hyperintensity along the corticomedullary junction, recurrent encephalitic-like episodes, peripheral neuropathy, or autonomic symptoms favors NIID (Sone et al., 2016). Cerebrotendinous xanthomatosis (CTX) should also be considered because it is a treatable metabolic leukodystrophy and may present with cerebellar and brainstem pathway involvement; cataracts, chronic diarrhea, tendon xanthomas, and elevated cholestanol are useful distinguishing clues (Nie et al., 2014; Björkhem, 2013). In the present case, the combination of long-standing bladder and bowel dysfunction, slowly progressive spastic-ataxic syndrome, symmetric frontoparietal white matter involvement, corticospinal tract and middle cerebellar peduncle hyperintensities, spinal cord atrophy, and molecular confirmation of an LMNB1 duplication strongly supported the diagnosis of ADLD.

**Table 1:** Differential diagnosis of leukoencephalopathies featuring middle cerebellar peduncle lesions.

Disorder	Key imaging feature	Key distinguishing features
LMNB1-related ADLD	Frontoparietal white matter lesions; infratentorial involvement of the brainstem corticospinal tract and middle cerebellar peduncles; spinal cord atrophy	Adult onset; early autonomic dysfunction, especially bladder/bowel dysfunction and orthostatic symptoms; autosomal dominant inheritance; LMNB1 duplication/upstream deletion
LBSL	Cerebral white matter, corticospinal tract, brainstem/cerebellar pathway, middle cerebellar peduncles, and spinal cord involvement; possible lactate peak	Prominent sensory ataxia and spasticity; spinal cord dorsal column and lateral corticospinal tract involvement; DARS2 biallelic variants
CLCN2-related leukoencephalopathy	middle cerebellar peduncles and brainstem tract involvement; white matter abnormalities	Optic atrophy, retinal abnormalities, hearing symptoms, hypogonadism or male infertility; usually milder clinical course; CLCN2 biallelic variants
FXTAS	middle cerebellar peduncles, brainstem, splenial, and diffuse white matter abnormalities	Older age, usually male; action/intention tremor, ataxia, cognitive decline, neuropathy/autonomic symptoms; FMR1 premutation
NIID	Diffuse leukoencephalopathy with possible middle cerebellar peduncles involvement	Curvilinear DWI hyperintensity at the corticomedullary junction; recurrent encephalitic-like episodes; neuropathy, myopathy, autonomic symptoms; NOTCH2NLC GGC repeat expansion
CTX	Cerebellar/brainstem pathway and white matter involvement	Treatable disorder; cataracts, chronic diarrhea, tendon xanthomas, dentate nucleus/cerebellar involvement; elevated cholestanol; CYP27A1 variants

ADLD, autosomal-dominant adult-onset demyelinating leukodystrophy; LBSL, Leukoencephalopathy with brain stem and spinal cord involvement and lactate elevation; FXTAS, Fragile X-associated tremor ataxia syndrome; NIID, Neuronal intranuclear inclusion disease; CTX, Cerebrotendinous xanthomatosis.

Within the broader differential diagnosis of adult-onset leukodystrophies, attenuated metabolic disorders—particularly lysosomal storage disorders (LSDs)—should also be considered. Recent studies have shown that highly attenuated LSDs may remain unrecognized until adulthood because they often present with nonspecific, organ-limited, or isolated neurological manifestations, leading to substantial phenotypic overlap with both inherited and acquired leukoencephalopathies (Urizar et al., 2025). This overlap is particularly relevant to *LMNB1*-related ADLD, as certain attenuated lysosomal leukodystrophies may likewise present with slowly progressive spasticity, ataxia, and white matter abnormalities. However, ADLD can usually be distinguished by the early prominence of autonomic dysfunction, its characteristic MRI pattern of frontoparietal white matter involvement, spinal cord atrophy, the “Beagle sign” caused by involvement of the brainstem and middle cerebellar peduncles, and an autosomal dominant family history associated with *LMNB1* copy-number alteration. Recognizing this overlap may improve the differential diagnosis of adult-onset white matter disease and help prompt appropriate metabolic and genetic testing.

With the continuous advancement of genetic testing technologies, the diagnosis of ADLD is increasingly dependent on genetic testing. However, neuroimaging remains a critical preliminary tool, particularly for the early identification of potential cases prior to genetic testing. Early recognition not only facilitates symptom management but also expands the window for clinical intervention. When such characteristic cranial and spinal magnetic resonance imaging findings coexist with symptoms of autonomic dysfunction—such as urinary incontinence, constipation, orthostatic hypotension, among others—clinicians should strongly suspect *LMNB1*-related ADLD and promptly proceed with genetic testing for confirmation.

This case demonstrates the typical clinical presentation and imaging characteristics of ADLD, highlighting the critical role of comprehensive clinical neurological examination and neuroimaging in its recognition and diagnosis. In particular, the “Beagle Sign” is proposed here as an illustrative imaging analogy that may raise clinical suspicion of *LMNB1*-related ADLD prior to genetic confirmation.

Several limitations of this report warrant consideration. First, the proposed “Beagle sign” is derived from a single-case observation. Although its constituent anatomical components are consistently present in the existing ADLD literature, they have not been previously conceptualized as a unified imaging pattern. Second, its diagnostic accuracy and inter-observer reliability lack systematic validation. Consequently, the “Beagle sign” should currently be viewed as a preliminary heuristic tool to facilitate pattern recognition, rather than a definitive diagnostic marker. Future collaborative, multicenter studies are needed to substantiate its true clinical utility and specificity.

## 4 Patient perspective

Our patient and his family expressed that obtaining a definitive genetic diagnosis provided “significant psychological relief” after years of uncertainty regarding the cause of his progressive symptoms. They acknowledged that while there is currently no curative therapy for ADLD, the diagnosis helped them plan for future care and understand the inheritance risk for the next generation.

## Data Availability

The original contributions presented in the study are included in the article/supplementary material, further inquiries can be directed to the corresponding author/s.
